# Gene Expression Profiling Indicated Diverse Functions and Characteristics of Core Genes in Pea Aphid

**DOI:** 10.3390/insects11030186

**Published:** 2020-03-15

**Authors:** Ruizheng Tian, Yixiao Huang, Balachandar Balakrishnan, Maohua Chen

**Affiliations:** Northwest A&F University, State Key Laboratory of Crop Stress Biology for Arid Areas, Key Laboratory of Integrated Pest Management on Crops in Northwestern Loess Plateau, Ministry of Agriculture and Rural Affairs, Yangling 712100, China; tianruizheng@nwafu.edu.cn (R.T.); yixiaohuang@nwafu.edu.cn (Y.H.); b.balachandar1988@gmail.com (B.B.)

**Keywords:** pea aphid, gene expression profiling, core genes

## Abstract

The pea aphid is a global insect pest, and variable phenotypes can be produced by pea aphids in the same genotype in response to changes in external environmental factors. However, detailed dynamic gene regulation networks and the core markers involved in different biological processes of pea aphids have not yet been reported. In this study, we obtained the published genomic and transcriptomic data, and performed transcriptome profiling of five pea aphid morphs (winged asexual female, wingless asexual female, wingless sexual female, winged male and wingless male) from each of three pea aphid genotypes, i.e., the transcriptomes from a total of 15 types of pea aphids were analyzed and the type-specific expression of genes in five different morphs was identified. The expression profiling was verified by quantitative real-time PCR (qPCR) analysis. Moreover, we determined the expression features and co-expression networks of highly variable genes. We also used the ARACNe method to obtain 263 core genes related to different biological pathways. Additionally, eight of the identified genes were aligned with transcription factor families, indicating that they act as transcription factors and regulate downstream genes. Furthermore, we found reliable markers using random forest methodology to distinguish different morphs of pea aphids. Our study provides a systematic and comprehensive approach for analyzing the core genes that may play important roles in a multitude of biological processes from the insect transcriptomes.

## 1. Introduction

The pea aphid *Acyrthosiphon pisum*, which depresses crop production and acts as a vector of various plant viruses, is a serious insect pest of multiple plants [1,2]. It has a complex life history, reproductive patterns and highly plastic phenotypes [3]. Multiple discrete phenotypes are produced by pea aphids with the same genotype in response to changes in external environmental factors such as temperature, light, host plants, symbiotic bacteria [4,5,6]. Aphid phenotypic plasticity, mainly including color plasticity and wing plasticity, is important for their adaptation to environmental and ecological changes. For example, parthenogenetic female aphids can produce winged or wingless offspring under different environmental conditions [7]. Studying these environment-related polyphenisms can lead to finding important genes related to environmental stress, which is of great significance for entomological research [5].

The formation of winged and wingless patterns of pea aphids was determined by both environmental conditions and the genotype of the aphid itself [8]. The occurrence of winged aphid directly affects the release efficiency of its natural enemies. Therefore, an in-depth study on the causes of aphid polyphenisms is of great importance for the biological control of this insect.

At present, the mechanisms of pea aphid phenotypic plasticity are complex and unclear [9,10]. Some studies have shown that the polyphenisms differences among some clones are related to host plants; however, it has also been found that different clones on the same host plant have diverse abilities to produce winged aphids [11]. In addition, in some aphid species, the different wing type characteristics of male aphids are mainly determined by genetic factors. For example, in the pea aphid, wing polymorphism is controlled by an allele (Api locus) on X chromosome, and individuals carrying api^w^ gene are wingless in general, while those carrying api^wl^ gene are apt to have wings [12]. Parthenogenetic aphids produced by female aphids exposed to short sunlight periods showed different wing types due to different genotypes of female aphids [13].

Therefore, determining the dynamic gene expression analysis in a diversity of phenotypes is essential for understanding the evolution of aphids [14]. Previous research has discovered morph-biased genes and measured the rates of evolution [4]. However, detailed dynamic gene regulation networks and the core markers involved in different biological processes of pea aphids have not yet been reported. Here, we analyzed the transcriptome data of five morphs from each of the three pea aphid genotypes. Similar to the former study [4], we also used the ‘tau’ value [15] to estimate the expression levels of each gene and analyze the gene expression features of each group, which provide preliminary data for our subsequent analyses. Furthermore, gene function annotation and enrichment analysis for each functional group was performed using Blast2GO, which could show the diverse functions occurred in different genotypes and morphs [16].

Meanwhile, we used the ARACNe algorithm to calculate the relationships among each gene pair and identified 263 core genes [17], which are critical node genes in the gene co-expression network and often perform important biological functions. Since there is little information on transcription factors in pea aphids, we obtained the protein sequences of core genes from the UniProt database (https://www.uniprot.org/) and aligned them to the transcription factor domains [18]. We obtained eight potential transcription factors that play roles in DNA replication, recombination and repair or in RNA processing and modification. Furthermore, we constructed a gene co-expression network using the core genes obtained by ARACNe and used a random forest algorithm to find prominent markers during the evolution of different pea aphid morphs [19].

## 2. Materials and Methods

### 2.1. Data Sources and Data Preprocessing

All of the RNA-seq data were downloaded from GEO datasets, with the accession number GSE56830. Fifteen types of RNA-seq data were mapped to the *A. pisum* genome (a version Acyr 2.0 which was indicated as an update in May 2017 in Aphidbase) by tophat2 (v2.1.0) [20]. Transcripts were assembled by cufflinks (v2.0.2) with the reference Acyr_2.0.38 downloaded from BioInformatics Platform for Agroecosystem Arthropods (BIPAA) (http://bipaa.genouest.org/is/aphidbase/) [21]. The quality trimming of data was performed by QTrim [22].

### 2.2. Identifying Specifically Expressed Genes

We applied the ‘tau’ value to calculate the expression level of type-specifically expressed genes [15]. The ‘Tau’ value was defined as:

$$\mathrm{tau}=\frac{\sum {}_{i=1}^{N}(1-x_{i})}{N-1},$$

where N is the number of types and X_i_ is the expression of the gene in the *i*-th sample divided by the highest expression among all samples of this gene. We defined genes with ‘tau’ values over 0.8 as type specifically expressed genes. 

### 2.3. Data Analysis and Gene Function Annotation

Data were analyzed by R software (version 3.4.0). We used blast2GO, which is a bioinformatics platform for high-quality protein function prediction and functional analysis, to align candidate genes with known annotated genes [16].

### 2.4. Insect Materials and Quantitative Real-Time Pcr (Qrt-Pcr)

All of the pea aphid samples were reared in cages with Vicia faba at 18 ± 1 °C; they had a photoperiod of L16:D8 and relative humidity of 60 ± 5%. To obtain the five morphs of pea aphids, we provided different conditions for the samples according to a previously published method [23]. We isolated total RNA from adult individuals (10 mg) of each morph. RNA extraction was performed with an RNAiso Plus kit (Takara Biotechnology, Dalian, China). The pea aphid *β-actin* was used as an internal control (Table S1). A PrimeScriptTM II 1st Strand cDNA Synthesis kit (Takara Biotechnology, Dalian, China) and SuperReal PreMix Color (SYBR Green (Tiangen Biotech, Beijing, China) kit was used for first-strand complementary DNA synthesis and qRT-PCR, respectively. All of the primers for qRT-PCR were designed using Primer Premier 6.0 software (http://www.premierbiosoft.com/primerdesign/). Each sample contained three biological replicates, and the relative expression of the 11 genes was calculated with the 2^−ΔΔCt^ method.

### 2.5. Gene Expression Patterns and Potential Transcription Factors

Mev software was used to obtain gene expression patterns. First, we normalized the gene expression using the input. Then, genes were clustered using the k-means clustering (KMC) method in the Mev software. Gene co-expression network was built using the MI scores calculated by the ARACNe method [17]. Genes with 20 or more co-expressed genes, of which the average MI score was over 0.4, were defined as core genes. The protein sequences of core genes were downloaded from the UniProt database. The function annotation of core genes was identified using the OrthoDB database (release 10) [24]. These protein sequences were aligned to known transcription factor domains using the Animal Transcription Factors Database (Animal TFDB) [25].

### 2.6. Random Forest Method to Find Morph-Biased Markers

The random forests (a classifier that combines many single decision trees) methodology was used to calculate the importance of each gene for defining different aphid types [19]. Genes that scored in the top 30 by the mean Gini importance of the model were used to classify each aphid type individually.

## 3. Results

### 3.1. Transcriptome Profiling of 15 Types of Pea Aphids

The transcriptomes of 15 types of pea aphids were downloaded from the Gene Expression Omnibus (GEO) database (https://www.ncbi.nlm.nih.gov/geo/) with the accession number GSE56830, which included the transcriptome data of five morphs (winged asexual female, WF; wingless asexual female, UWF; wingless sexual female, SF; winged male WM; and wingless male, UWM) from three pea aphid genotypes (F1, I18, and Bk11) were used for analyses. In other words, transcriptome data for 15 pea aphid types (F1_WF, I18_WF and Bk11_WF; F1_UWF, I18_UWF and Bk11_UWF; F1_SF, I18_SF and Bk11_SF; F1_WM, I18_WM and Bk11_WM; F1_UWM, I18_UWM and Bk11_UWM) were used. 

**Figure 1:**
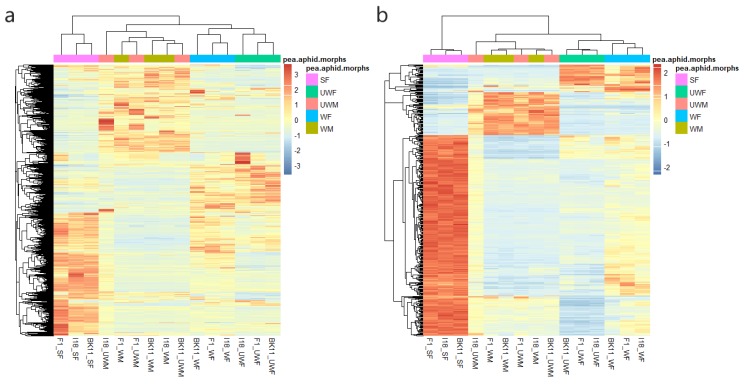
Gene expression profiling and gene functions of 15 types of pea aphids. Heatmap clustering of total (**a**) expressed genes and (**b**) sample specifically expressed genes showing hierarchical relationships based on gene expression similarity. F1, I18, and Bk11 each represent a genotype. Winged asexual female, WF; wingless asexual female, UWF; wingless sexual female, SF; winged male WM; and wingless male, UWM.

After removing low-quality reads, 1384 million reads remained (Table 1). These reads were aligned to the *A. pisum* genome (Acyr 2.0) by tophat2 (v2.1.0) and assembled by cufflinks (v2.0.2). To investigate the gene expression patterns in the 15 types of pea aphids, we identified 25,585 expressed genes with the FPKM (Fragments Per Kilobase per Million cutoff) > 0.5. After clustering all of the genes expressed by the 15 pea aphids, we observed dynamic regulation in the distinct types (Figure 1a). It is interesting that samples with the same sex were confirmed to be clustered together.

Meanwhile, many genes were differentially expressed among the variable pea aphid types. To discover the type-specifically expressed genes among the five morphs, we measured the specific level using ‘tau’ value. As a result, 418 type-specifically expressed genes were identified with the ‘tau’ value over 0.8 (Table S2, Figure 1b). Most of those genes were specifically expressed in the sexual female samples.

**Figure 2:**
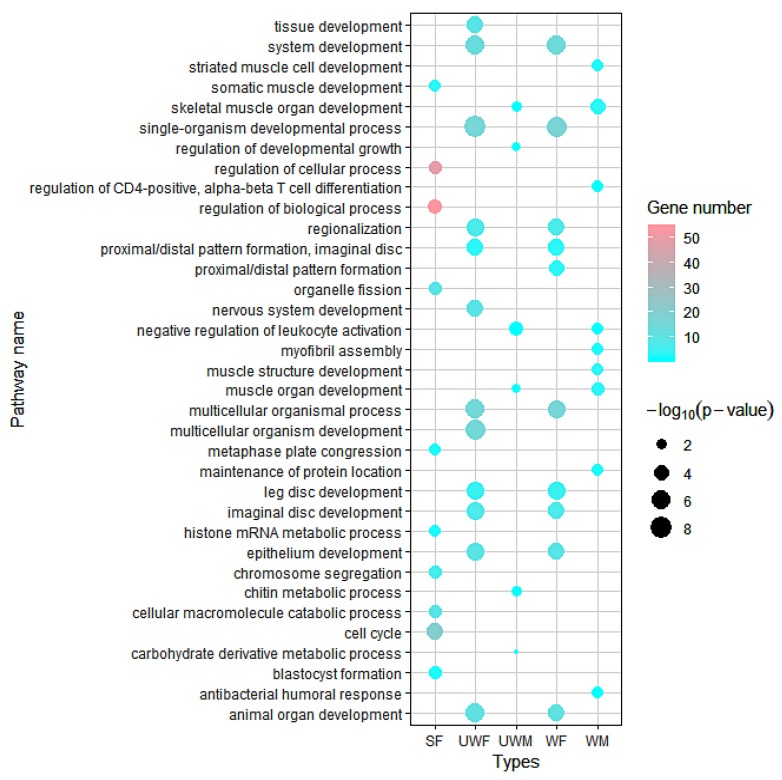
Gene functions of type-specifically expressed genes in five pea aphid morphs (wingless sexual female, SF; wingless asexual female, UWF; wingless male, UWM winged asexual female, WF; and winged male WM) from each of the three pea aphid genotypes (F1, I18, and Bk11). GO terms of type-specifically expressed genes were enriched using Blast2GO. 3.2. Confirmation of screened gene expression.

To discover the gene functions of type-specifically expressed genes, we clustered the gene ontology (GO) terms for them and found varied functions of the type-specifically expressed genes among the five morph types (WF, UWF, SF, WM and UWM) (Figure 2). Tissue development pathways were found in the ‘WF’ and ‘UWF’ groups, and immune-related pathways were found in the ‘WM’ and ‘UWM’ groups. Additionally, we also observed cell cycle and chromosome segregation pathways in ‘SF’ samples. Overall, there were diverse features of gene expression levels and functional pathways among different types of pea aphids. 

**Figure 3:**
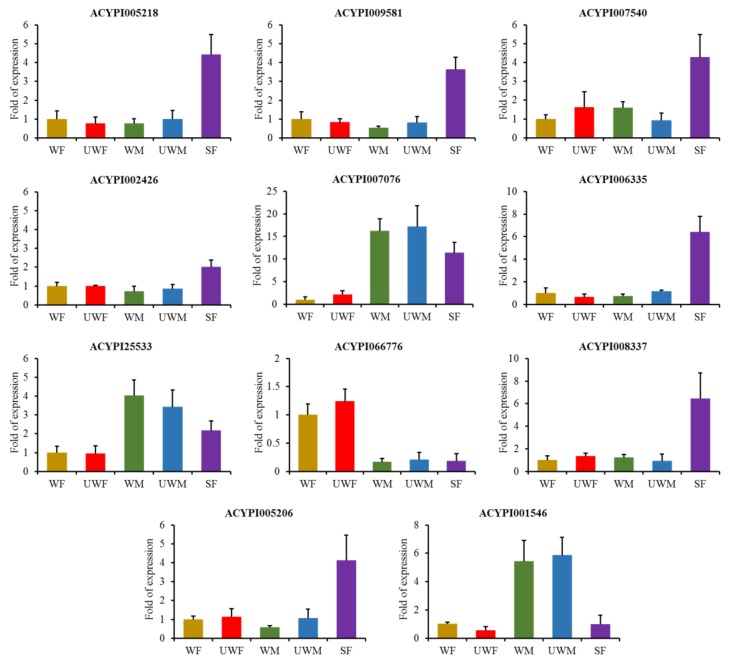
Expression levels of 11 selected type-specifically genes in five morphs of pea aphids. The *β-actin* gene was used as internal references to normalize target gene expression. Data are shown as means ± SE of three replicates.

To support the expression profiling determined by RNA-seq data using the ‘tau’ value, all 11 type-specifically expressed genes were selected for qPCR analysis (Figure 3). RNA samples were isolated from the pea aphid individuals of each morph (Figure S1a). The melting curve analyses were used to confirm the primer pairs for specific PCR products (Figure S1b). The expression patterns of 11 genes in different morphs of pea aphids were consistent with the RNA-seq expression details (Figure 3, Table S2). The transcripts of ACYPI007076, ACYPI25533 and ACYPI001546 were abundant in the winged and wingless male morphs. The expression level of ACYPI066776 was the highest in winged and wingless asexual female aphids. The other seven genes, ACYPI005218, ACYPI009581, ACYPI007540, ACYPI002426, ACYPI006335, ACYPI008337 and ACYPI005206, were highly expressed in sexual female individuals.

### 3.2. Gene Expression Patterns and Potential Transcription Factors

**Figure 4:**
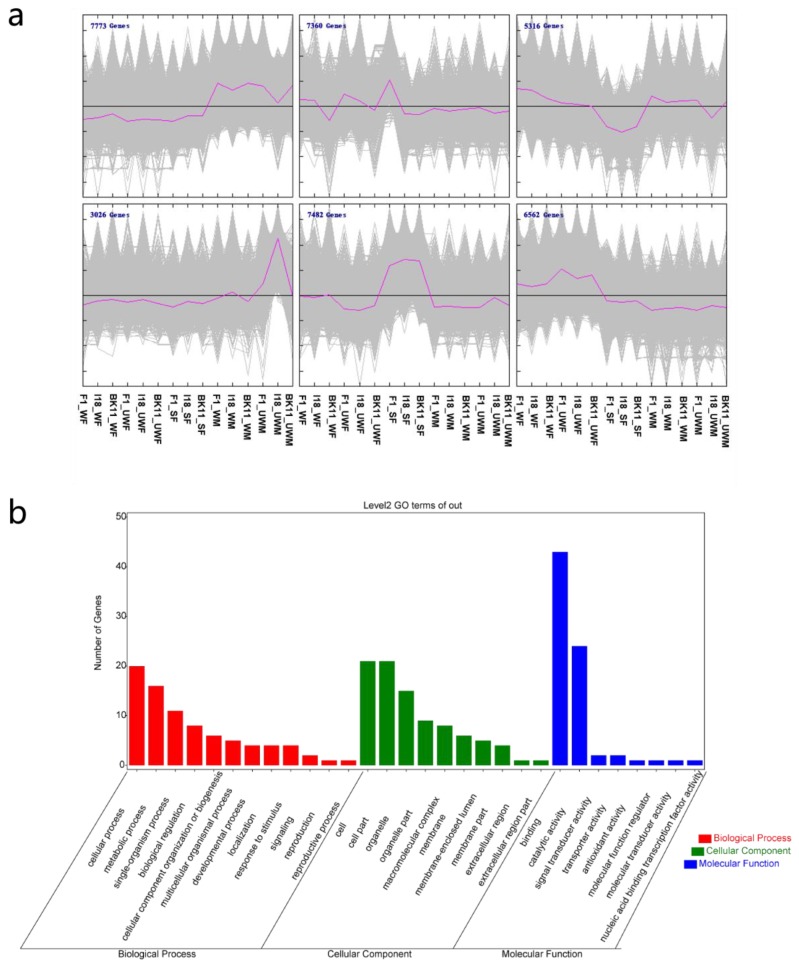
Dynamic gene expression regulation and functional annotation of core genes. (**a**) Six dynamic gene expression patterns during 15 different types; (**b**) biological pathway, molecular function and cell component categories for core genes.

Furthermore, to show the dynamic regulation network of gene expression of different aphids, we divided the gene expression pattern into six types using Multiple Experiment Viewer (Mev) software (Figure 4a). A total of 7482 genes were highly expressed in sexual female samples, whereas 5316 genes tended to be expressed at low levels in sexual female samples. We also found that there were some genes specifically expressed in the three different genotypes. For example, 3026 genes were highly expressed only in I18 genotypes of ‘UWM’ samples, indicating the complexity and diversity of aphid gene expression.

Due to the complex interactions involved in gene regulation, we aimed to identify co-expressed genes, which would uncover the core genes during phenotype alteration. First, genes with a standard deviation over 6 were considered to be highly variably expressed genes. Subsequently, the ARACNe method was applied to calculate the correlation score of each interacted gene. 

As a result, 263 genes were selected as potential core genes with at least 20 co-expressed genes, and the average mutual information (MI) score was greater than 0.4. The clustered core genes were involved in the single-organism process, developmental process, signaling, reproduction, and so on (Figure 4b). We identified possible transcription factor of these 263 core genes, and found eight transcription factor which belonged to the HTH, THAP, zf-H2C2_2, and CSRNP_N transcription factor families (Table 2). Among these, ACYPI010241, ACYPI28272 and ACYPI23901 were related to DNA replication, recombination and repair, as determined by the OrthoDB database, whereas ACYPI088029 might participate in RNA processing and modification. In addition, ACYPI080640 might be involved in regulating cell cycle control, cell division, and chromosome partitioning. These results demonstrated that these potential transcription factors played substantial roles in various important biological pathways and evolution in pea aphids.

### 3.3. Gene Co-Expression Network and Morph-Biased Markers

**Figure 5:**
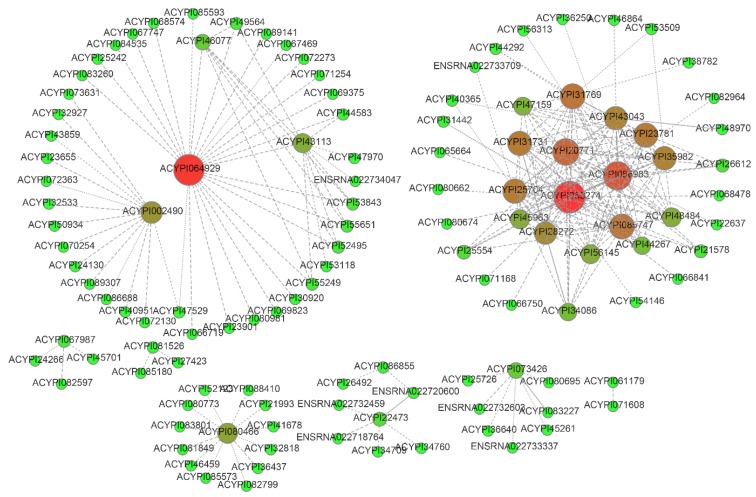
Gene co-expression network of highly variable expressed genes. Colors reflect connectivity: green represents low connectivity, and red represents high.

To better display gene interaction relationships, we used the interacted genes with mutual information (MI) score over 0.5 to construct a gene co-expression network. As displayed in Figure 5, there were two major groups in the network. Functions of some core genes were clearly annotated, for example, the core gene ACYPI064929 had membrane function in cellular component of GO, and ACYPI063274 was homologous with one of the Wiskott–Aldrich syndrome protein family members in humans. Other prominent genes were also important in the biological process of pea aphids. For example, ACYPI085747 and ACYPI20771 were related to ATP synthesis coupled proton transport and proteolysis, respectively. ACYPI31769 could act as an ATP binding protein and take part in microtubule motor activity. 

**Figure 6:**
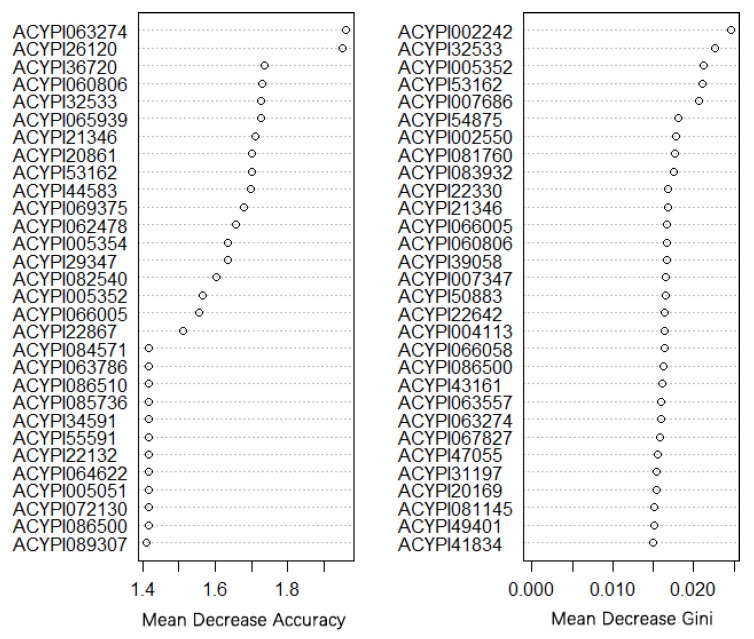
The score of mean decrease accuracy and mean decrease Gini in top 30 marker. The top 30 genes (mean Gini importance) were identified by the random forest model when classifying all sample types.

Furthermore, considering that some redundant and irrelevant features might influence the classification of different morphs, we used random forest algorithm to cluster the gene types by the morphs of each sample and identified the top 30 (mean Gini importance) markers, which could be used to predict the morph of pea aphid by gene expression level (Figure 6). Mean decrease Gini, which represents the performance of fit (it measures the importance of each predictor in dividing data into different classes) and mean decrease accuracy, which reflects accuracy in the model prediction (it measures how much each factor reduces the error of classification in the model), were used in our analysis. The detailed ‘mean decrease accuracy’ and ‘mean decrease Gini’ of each gene are shown in Table S3. Among them, ACYPI32533, ACYPI060806, ACYPI21346, ACYPI53162, and ACYPI005352 obtain high marks for both the mean decrease accuracy score and the mean decrease Gini score. ACYPI060806 is a member of the zinc finger C2H2 superfamily and participates in nucleic acid binding. ACYPI21346 and ACYPI53162 are integral components of the membrane. In addition, ACYPI005352 contains a double-stranded RNA-binding domain, which may participate in transcription. These crucial functions may be responsible for the classification of those genes. 

## 4. Discussion

Insects often display an extreme form of phenotypic plasticity called polyphenism, in which two or more distinct morphs can develop from the same genotype [26,27]. Phenotypic plasticity shows the maximum adaptability of species to environmental changes. The study of phenotypic plasticity is useful for a comprehensive understanding of the evolution of insect life history and provides important help for the effective management of insect pests [5]. Although there have been many studies on the morphology, physiology and life history of aphid wing differentiation [28,29,30,31], few studies have been conducted at the transcriptome and genome levels. 

Purandare et al. (2014) have done an excellent job of phenotypic plasticity and performed important analysis on rates of evolution. The data provided by Purandare et al. (2014) facilitated further analyses in the mechanisms of pea aphid phenotypic plasticity. Based on the genomic and transcriptomic data from the public database, we did tau analysis to identify the gene expression specificities and gene functions of 15 types of pea aphids. In comparison to the analyses by Purandare et al. 2014, we focused on the detailed specifically expressed genes and their function, and further verified the expression of those genes using qPCR, but not on the rates of evolution between morph-biased genes and ubiquitously expressed genes. Our work contributed some new results to the diverse functions and characteristics of the core genes in the pea aphid, which includes: (i) the gene co-expression network which can reflect the interaction and regulatory networks of morph related genes; (ii) core genes which may play a vital role in the regulation of gene expression, and eight of them were identified as potential transcription factors; (iii) qPCR confirmation of the expression of some specifically expressed genes; (iv) morph-biased marker genes which can be used for further aphid polyphenisms research.

To uncover the internal gene regulation mechanism, we described the transcriptome profiling and gene expression features of different types of pea aphids. The work in Purandare et al. (2014) performed tau analysis and connected it with rates of evolution. In our study, we used a similar tau analysis method to identify the especially expressed genes, which was the base and first step for our further analyses focusing on the detailed core genes, as well as the functions analyses and expression confirmation by qPCR for those genes. From the 418 type-specifically expressed genes obtained in the analysis, we identified 263 core genes that were classified according to their GO terms using Blast2GO, and found that these core genes tend to take part in response to stimuli, signaling, reproduction, and so on. These pathways might play important roles in aphid differentiation. 

Our study provided a new view of insect transcriptome data mining, especially those which lack enough information on gene annotation. However, many genes remain hard to explain due to the genome specificity of the pea aphid. These genes need to be identified using molecular biology methods, such as qPCR and immunofluorescence technique. Fortunately, most genes in our study can be evaluated and classified using type-specifically expression method and gene co-expression method.

The work in Purandare et al. (2014) identified morph-biased and unbiased genes, as well as morph-specific genes using Deseq2 and tau analysis. We did further analyses based on that work and our tau analysis, and found that aphid-specific expressed genes in SF were significantly enriched in the cell cycle pathway according to our functional annotation of morph-specifically expressed genes. On the other hand, the aphid-specific expressed genes were found in samples of the same sex, instead of the same wing type, which tended to have similar functions. Morph-specific functions of samples from different sexes are very different, indicating that the polyphenisms differences between genders differ greatly in comparison with differences between wing types. Female aphids express more organ differentiation-related genes, while male aphids express more genes participating in T cell differentiation and leukocyte activity (Figure 2). Among the 11 genes which we validated using qPCR (Figure 3), ACYPI007076, ACYPI25533 and ACYPI001546 are highly expressed in male aphid. Among them, ACYPI007076 participates in the ATP binding and ACYPI007076 pathways, and it contains the immunoglobulin-like domain, which may participate in the immune pathway. ACYPI25533 participates in covalent chromatin modification and covalent chromatin modification, while ACYPI001546 is related to posttranslational modification and transcription. In addition, we verified that ACYPI066776, which is highly expressed in female aphids, might participate in nucleic acid binding.

Due to data limitations, we could hardly discover other biological mechanisms of pea aphids. The former study of this dataset demonstrated the morph-related genes of aphids. We applied the ‘tau’ value to estimate the gene specifically expressed possibilities, which could accurately reveal the gene specificity. In comparison to the work in Purandare et al. (2014), we enriched the gene functions of all the type-specifically expressed genes in SF, UWM, WM, UWF, and WF. The type-specifically expressed genes were classified into GO terms such as tissue development, T cell differentiation, and cell cycle. To further investigate the gene co-expression network of aphid differentiation, we used ARACNe to calculate the MI scores of each gene pair, which is more effective than the normal ‘person correlation’ method and can represent different correlations of each pair of interacted genes and the importance of each individual gene. The expression of the 263 core genes varied significantly among different aphid morphs, and these core genes had high expression correlations with other genes. Core genes could act as hub genes in the gene expression regulatory network during aphid differentiation. We found that eight of the core genes were aligned to different transcription factor families. This work helps biologists to better understand the gene expression regulation of pea aphids. The eight potential transcription factors have HTH, THAP, zf-H2C2_2, and CSRNP_N domains and can participate in DNA replication, chromosome partitioning, cell cycle pathways and RNA processing and modification pathways [32,33,34,35], indicating crucial and diverse roles in gene expression network.

The markers identified by random forest algorithm can act as classifiers for the research of phenotypic plasticity in pea aphids, especially those with high Gini scores, such as ACYPI002242, which is related to metal ion binding, ACYPI32533, which is currently unannotated, and ACYPI005352, which has a double-stranded RNA-binding domain. Those genes with high Gini scores must be validated by molecular biology experiments. In addition, the core genes, especially the transcription factors and the genes highly related to aphid differentiation detected by ARACNe or random forest algorithms, are likely involved in multiple biological processes and are worth further investigation. The random forest method in our analysis provided further evidence of the phenotypic plasticity-related genes identified in both the work by Purandare et al. (2014) and ours. In conclusion, we explored the transcriptome data at sufficient depth and found core markers in the dynamic regulation network of different types of pea aphids. These core markers are of great importance in aphid polyphenisms, and are worth to be studied as potential regulatory genes affecting phenotypic plasticity.

## 5. Conclusions

In this study, we provided an effective approach for analyzing the insect transcriptome and found gene expression rules and core genes using 15 types of pea aphid transcriptome dataset. We obtained 418 type-specifically expressed genes using gene expression profiling of pea aphid, and the qPCR results were consistent with the details from the expression profile. We obtained the gene expression feature of different aphid types, and found 263 potential core genes, which may play a vital role in the regulation of gene expression. We also identified eight potential transcription factors and top 30 morph-biased markers, providing the new idea and path for the study of transcription factors and phenotypic plasticity of the pea aphid. We presented a comprehensive framework for analyzing the insect transcriptome, and the excavated core genes may play a complex and crucial role in multiple biological processes of aphids.

## Figures and Tables

**Table 1:** Mapping rate of 15 different types of pea aphids.

	Total Reads	Mapped Reads	Mapping Rate
F1_WF	93097593	79389596	85.30%
F1_UWF	101313506	88512217	87.40%
F1_SF	91946734	82783997	90.00%
I18_WF	109616712	80872988	73.80%
I18_UWF	82776192	59534227	71.90%
BK11_WF	102979257	97275709	94.50%
BK11_UWF	106808567	96654529	90.50%
F1_WM	80133908	67200794	83.90%
F1_UWM	68817463	62643966	91.00%
I18_WM	100710783	63792363	63.30%
I18_UWM	94077934	63733175	67.70%
I18_SF	106615474	92694245	86.90%
BK11_WM	82734259	70391904	85.10%
BK11_UWM	71796221	66195361	92.20%
BK11_SF	90793111	87397909	96.30%

**Table 2:** Alignment of transcription factor domains in eight core genes.

Gene ID	Family	Full Sequence e-Value	Best Domain e-Value	Domain Number
ACYPI006621	HTH	1.70 × 10^−10^	4.00 × 10^−10^	1
ACYPI010241	HTH	4.70 × 10^−07^	1.10 × 10^−06^	1
ACYPI088029	THAP	2.90 × 10^−19^	4.10 × 10^−19^	1
ACYPI089479	THAP	2.00 × 10^−05^	5.00 × 10^−05^	1
ACYPI28272	zf-H2C2_2	1.80 × 10^−112^	2.60 × 10^−10^	16
ACYPI23901	zf-H2C2_2	6.50 × 10^−92^	3.90 × 10^−11^	14
ACYPI29290	zf-H2C2_2	1.10 × 10^−32^	3.30 × 10^−10^	6
ACYPI080640	CSRNP_N	9.40 × 10^−06^	1.00 × 10^−05^	1

## Supplementary Materials

The following are available online at https://www.mdpi.com/2075-4450/11/3/186/s1, Figure S1: Electropherograms of 15 RNA samples used for qRT-PCR and melting curves of two genes. (a) Electropherograms of RNA extracted from the five morph types (from left to right, WF, UWF, SF, WM and UWM, all types contain three replications and the fifth lane is Marker DL2000). (b) The melting curves of qRT-PCR results of β-actin (left) and ACYPI006335 (right), and the melting curve of β-actin contains standard values. Table S1: The primers designed for quantitative real-time PCR, Table S2: Gene expression and tau values of type-specifically expressed genes, Table S3: Random forest results based on gene expression.
