# Supplementary figures and images for: Gene Expression Profiling Indicated Diverse Functions and Characteristics of Core Genes in Pea Aphid
Source: Insects. 2020 Mar 15;11(3):186. doi: 10.3390/insects11030186 (PMC7142545; doi:10.3390/insects11030186)

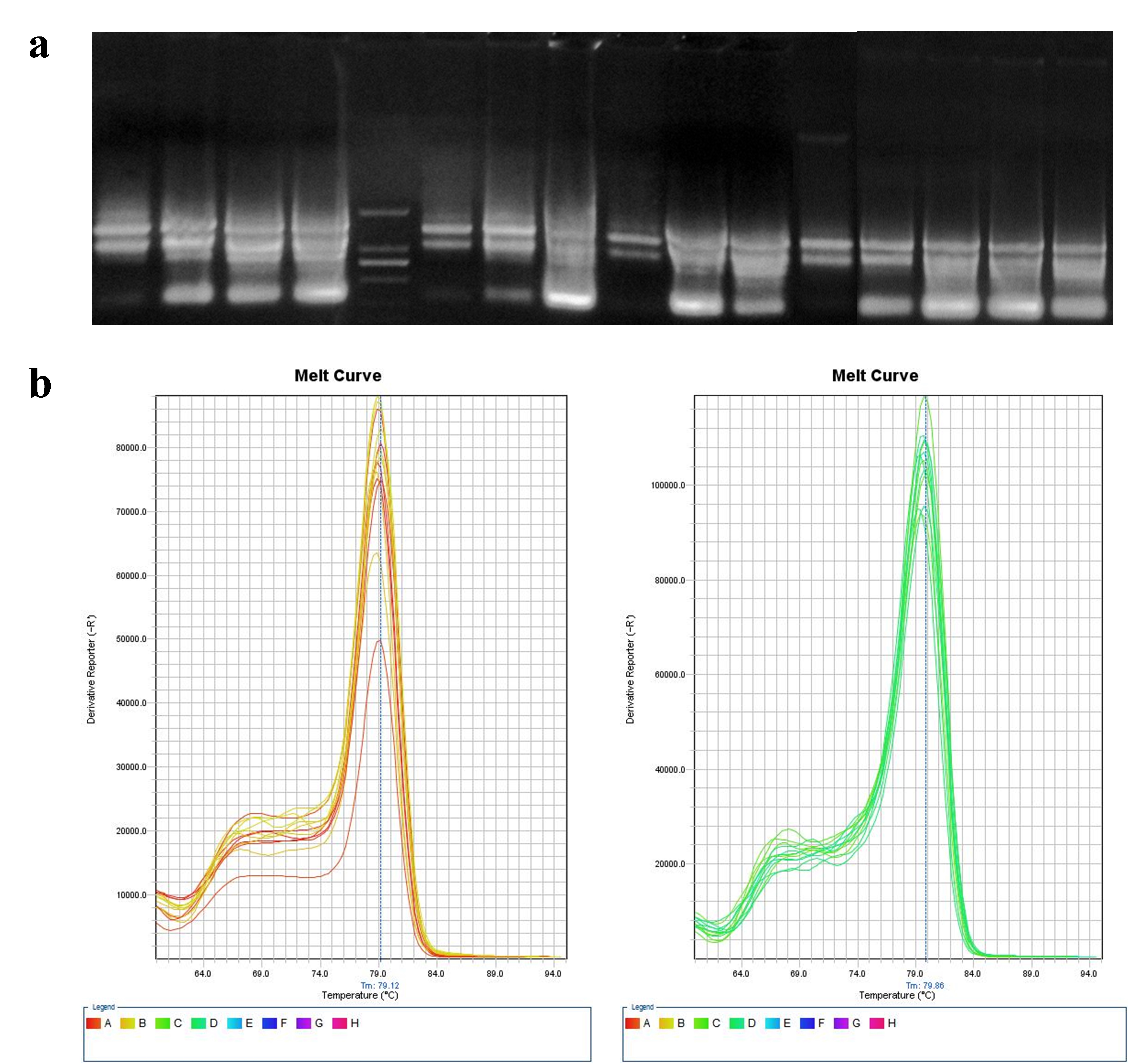

Supplement: Supplementary file 1 [file insects-11-00186-s001.zip › Supplementary_materials/Figure-S1-15Mar2020.jpg]
